# Effect of Moderate Beer Intake on the Lipid Composition of Human Red Blood Cell Membranes

**DOI:** 10.3390/nu16203541

**Published:** 2024-10-18

**Authors:** Anallely López-Yerena, Natalia Muñoz-García, Victoria de Santisteban Villaplana, Teresa Padro, Lina Badimon

**Affiliations:** 1 Institut Recerca Sant Pau, Sant Antoni Maria Claret 167, 08025 Barcelona, Spain; naye.yerena@gmail.com (A.L.-Y.); nmunoz@santpau.cat (N.M.-G.); vsantisteban@santpau.cat (V.d.S.V.); tpadro@santpau.cat (T.P.); 2 School of Pharmacy and Food Sciences, University of Barcelona (UB), 08036 Barcelona, Spain; 3 Centro de Investigación Biomédica en Red Cardiovascular (CIBER-CV), Instituto de Salud Carlos III, 28029 Madrid, Spain; 4 Cardiovascular Research Chair, Universitat Autònoma de Barcelona (UAB), 08193 Barcelona, Spain

**Keywords:** erythrocytes composition, phospholipids, fatty acids, cholesterol, alcohol consumption

## Abstract

**Background/Objectives**: Growing evidence suggests that erythrocyte membrane lipids are subject to changes during their lifespan. Factors such as the type of dietary intake and its composition contribute to the changes in red blood cell (RBC) membranes. Due to the high antioxidant content of beer, we aimed to investigate the effect of moderate beer consumption on the lipid composition of RBCs membranes from healthy overweight individuals. **Methods**: We conducted a four-weeks, prospective two-arm longitudinal crossed-over study, where participants (*n* = 36) were randomly assigned to alcohol-free beer group or traditional beer group. The lipids of RBCs membranes were assessed at the beginning and the end of the intervention by thin-layer chromatography. **Results**: Four-weeks of alcohol-free beer promoted changes in fatty acids (FA), free cholesterol (FC), phosphatidylethanolamine (PE) and phosphatidylcholine (PC) (*p* < 0.05). Meanwhile, traditional beer intake led to changes in FA, FC, phospholipids (PL), PE and PC (*p* < 0.05). The observed alterations in membrane lipids were found to be independent of sex and BMI as influencing factors. **Conclusions**: The lipid composition of erythrocyte membranes is distinctly but mildly influenced by the consumption of both non-alcoholic and conventional beer, with no effects on RBC membrane fluidity.

## 1. Introduction

Human erythrocytes or red blood cells (RBCs) are highly specialized cells derived from haemopoietic stem cells in bone marrow, that have lost all their organelles after a maturation process driven by erythropoietin [1,2]. Once in the bloodstream, the mature erythrocyte has a biconcave, discoid shape, and the lack of organelles confers this cell the flexibility needed to circulate in the cardiovascular system. Flexibility, or deformability, is an intrinsic characteristic of healthy RBCs, allowing them to travel through tiny capillaries and deliver oxygen to all tissues. Conversely, a decrease in their flexibility might trigger hemolysis in the capillaries and premature removal of RBCs by reticuloendothelial macrophages, which results in altered tissue oxygenation [3,4]. It is becoming increasingly clear that RBCs are involved in several biological processes, in addition to being transporters of O_2_ and CO_2_ between the lungs and peripheral tissues, which are of fundamental importance for cardiovascular function [5].

A key component of RBCs is the membrane, and its molecular composition seems to modulate the rheological properties of the cell, what might play an important role in the cardiovascular pathophysiology. The human RBC membrane composition consists in lipids (41%), proteins (52%) and carbohydrates (7%). In the lipid bilayer, there is a complex mixture of different lipid classes: neutral lipids (NL, 25.2%, mainly cholesterol), phospholipids (PL, 62.7%) and glycosphingolipids (around 12%) [6]. Regarding PL, there are four major subclasses asymmetrically distributed in the lipid bilayer. The outer leaflet of the membrane is rich in phosphatidylcholine (PC] representing 27% of total membrane PL and sphingomyelin (SM) which represents 23%. The inner leaflet is mainly composed by phosphatidylethanolamine (PE, 30%) and phosphatidylserine (PS, 15%) [1].

Abnormalities in the molecular composition and biophysical properties of erythrocyte membranes have been associated with cardiovascular diseases [7], such as hypercholesterolemia [8], hypertension [9] and acute coronary syndrome [10,11,12]. These associations have been attributed to (i) alterations in the fluidity of RBC membranes due to changes in the PL, cholesterol and fatty acids (FA) composition [7,8,9] and (ii) destabilization of atherosclerotic plaques [10,11,12], disordered oxygen transport by hemoglobin [7], among others.

The lipid composition of erythrocyte membranes is subject to changes during their lifespan, which is approximately 120 days [13]. In fact, it is well known that RBCs can interact with lipoproteins in the bloodstream, resulting in lipid transfer and membrane remodeling. RBCs lack of the capacity to esterify cholesterol or store cholesteryl esters. Therefore, RBCs are a source of free cholesterol (FC) that can be rapidly exchanged with lipoproteins [14]. Moreover, diet can also modify the lipid composition of erythrocyte membranes. A previous study showed that Mediterranean diet induced changes in the lipid composition of erythrocyte membranes that influenced the structural properties of the cell [15]. However, the impact of diet, especially that of fermented alcoholic beverages, on the membrane composition of erythrocyte has been scarcely studied. Some previous studies have indicated that moderate alcohol consumption decreases lipid peroxidation in erythrocyte membranes and that this is due to changes in lipid composition [16]. Even so, the effects of alcohol consumption on erythrocyte membranes are poorly understood and there is a controversy regarding its impact on cardiovascular health.

Here, we designed a study to investigate the effect of moderate alcoholic and non-alcoholic beer consumption on the lipid composition of erythrocyte membranes from healthy individuals. For this purpose, a four-weeks prospective two-arm longitudinal crossed-over study was carried out in 36 overweight and obesity class 1 individuals.

## 2. Materials and Methods

### 2.1. Experimental Design and Study Population

Thirty-six healthy adult individuals between ages of 40–60 years, non-smokers, regular but moderate beer consumers (self-reported consumption), and with overweight (BMI, 28–29.9 kg/m^2^) or obesity class 1 (BMI, 30–35 kg/m^2^) were recruited [17]. Moderate beer drinking was defined according to the “Dietary Guidelines for Americans 2015–2020”, U.S. Department of Health and Human Services and U.S. Department of Agriculture, (https://www.niaaa.nih.gov/alcohol-health/, accessed on 5 November 2013, overview-alcohol-consumption/moderate-binge-drinking) and refers up to 1 drink per day for women, and up to 2 drinks per day for men.

**Figure 1:**
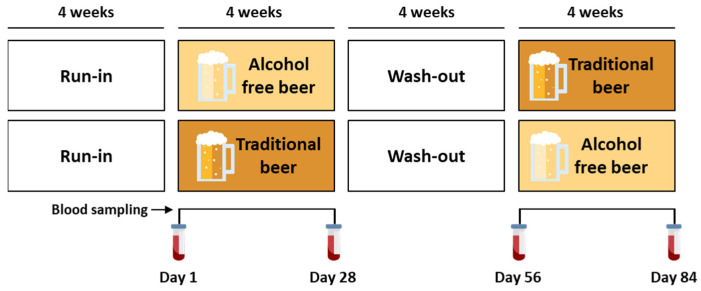
Experimental protocol for the intervention study.

The study was an open, randomized two-arm longitudinal cross-over trial with 4-week intervention periods (Figure 1). All subjects underwent two treatment sequences of 4 weeks, separated by a 4-week washout period, as previously described [17]. Initially, participants had a four-week period without beer (run-in). After this period, participants were randomly assigned to one of two interventions, each comprising 18 participants: non-alcoholic beer or traditional beer. After these four weeks of treatment, participants had washout phase (from day 28 to 56). This was followed by a second four-week period in which the groups exchanged their respective interventions. A simple randomization was performed using computer-generated random numbers. A random number and group were assigned to each subject at the moment of enrolment purely by chance.

Briefly, men drank two cans (660 mL beer) and women one can (330 mL beer) per day of alcohol-free beer (0.0 g alcohol) or traditional beer (15 g of ethanol, equivalent to 5.7% v) during the intervention periods. Traditional and alcohol-free beers were of the lager type from the same Spanish commercial brand.

Individual and total phenolic compound content of the beers administered to the volunteers is provided in Table S1 (Supplementary Information). The total phenolic content in alcohol free beer was 375.9 ± 157.0 mg/L and in traditional beer was 604.8 ± 190.0 mg/L. In both beers, ferulic acid and isoxanthohumol were the most abundant phenolic compounds.

During the intervention (run-in, wash-out periods and intervention phases), participants were not allowed to consume drinking alcoholic beverages and alcohol-free beer out of those provided as part of the study. Dietary patterns, assessed through the utilization of food frequency questionnaires, were documented before each visit, with infrequent alterations in dietary behaviors being reported. Adherence was assessed through routine phone communication with participants and conducting interviews at the conclusion of each intervention phase. Additionally, participants documented their daily beer consumption on a diary card. Furthermore, at the end of each intervention period, a clinician evaluated any potential side effects or symptoms such as facial flushing, abdominal discomfort, lightheadedness, emesis, diarrhea, which could be linked to the study interventions.

### 2.2. Ethical Statement

Informed written consent was obtained from all participants before entering the study and the participants were able to withdraw from the study at any time without giving a reason. The study was approved by the Human Ethical Review Committee of the Hospital “Santa Creu i Sant Pau” of Barcelona (Ref 14/186; 12 November 2014). All procedures were carried out in compliance with the principles outlined in the Declaration of Helsinki.

### 2.3. Sample Collection

Twelve hour fasting blood samples from the 36 volunteers were obtained at day 1 and 28 (first period) and day 56 and 84 (second period). Briefly, blood samples were collected in ethylene diamine tetra acetic acid (EDTA) or sodium citrate-containing vacutainer tubes for plasma and erythrocyte ghost preparation, respectively. Plasma was obtained after blood centrifugation at 1800× *g* for 20 min and preserved at −80 °C until used for lipoprotein extraction.

### 2.4. Human Erythrocyte Ghosts’ Preparation

Fresh blood from volunteers was collected in Vacutainer tubes containing sodium citrate. Tubes were centrifuged at 2500 rpm for 15 min and the plasma, and the buffy coat layer was carefully removed. Lack of hemolysis was visually controlled in all samples and those with signs of hemolysis were discarded. Cell membrane purification was performed immediately after.

A hypotonic solution (PBS 1X pH 7.4 + 14 mM EDTA) was added to the tubes and samples were homogenized. As previously described, additional centrifugation cycles with the hypotonic solution were performed until the buffy coat layer was completely removed. Consecutively, a second hypotonic solution (H_2_O Braun + 14 mM EDTA) was added to the tubes, which were left in agitation at 4 °C overnight. To remove the hemoglobin after hemolysis, further centrifugation (3220× *g*, 4 °C, 45 min) and homogenization steps were performed in the 2nd hypotonic solution until a cream-colored pellet was obtained. The resulting cream-colored pellets were further washed in shorter centrifuge cycles of 20, 15 and 10 min at 3220× *g* until the colorless ghosts were obtained. Thereafter, samples were transferred to Eppendorf tubes and stored at −80 °C under controlled conditions until their analysis.

### 2.5. Thin Layer Chromatography (TLC)

The lipid composition of lipoproteins was determined by thin layer chromatography (TLC) before and after the intervention. Lipids were extracted from erythrocyte ghosts using a dichloromethane/methanol (1:2 *v*/*v*) mixture and the organic solvent was removed under nitrogen stream. For the study of NL, lipid extracts were suspended in dichloromethane and applied to TLC glass plates (Macherey-Nagel TLC glass plates SIL G-25 UV254, 20 × 20 cm, Macherey-Nagel, France) along with different concentrations of lipid standards (a mixture of cholesterol, cholesterol palmitate, triglycerides (TAG), diglycerides and monoglycerides). For development (lipid separation), a mobile phase composed of heptane/diethyl ether/acetic acid (74:21:4, *v*/*v*/*v*) was used.

Additionally, a subgroup of 18 volunteers was randomly selected for further analysis using HPTLC to study changes in different phospholipid classes, specifically PS, PE, PC, and SM. The lipid extracts were suspended in a chloroform/methanol mixture (1:1 *v*/*v*) and applied to high-performance TLC (HPTLC) plates (HPTLC Silica Gel 60, F_254_, Glass plates, 20 × 20 cm, MilliporeSigma™, Madrid, Spain) along with different standards of PL (PE, PC, SM and PS). For development, a mobile phase composed of chloroform/methanol/ammonium hydroxide (65:25:4, *v*/*v*/*v*) was used. Finally, a staining solution composed of sulphuric acid, phosphomolybdic acid, and ethanol was applied to visualize the lipid bands, which were quantified by densitometry.

### 2.6. Anthropometric Data, Blood Pressure, Serum Lipid Profile and Other Biochemical Measurements

In addition, anthropometric measurements (body weight, waist circumference and BMI) were determined at baseline, before starting the intervention, and at the end of the intervention periods. The serum lipid profile such as total cholesterol, high density lipoprotein cholesterol (HDLc), nonHDL, low density lipoprotein (LDL), very-low density lipoprotein (VLDL) and TAG and other biochemical measurements were performed as previously described [17].

### 2.7. Statistical Analysis

Normality of distribution was assessed by the Shapiro-Wilk test. Differences in the baseline characteristics by sex and overweight vs. obesity were analyzed by Mann-Whitney test. To evaluate any differences at the end of the study with respect to the baseline in each arm group, a paired *t*-test for NL and Wilcoxon-test for PL subclasses were used. Differences in the changes between groups were analyzed by paired *t*-test for NL and Wilcoxon-test for PL subclasses. Correlation between RBCs lipids and blood lipids were assessed by means of Pearson’s correlation. All statistical analyses were conducted using STATA 15 (College Station, TX, USA) software. All reported *p*-values are two-sided, and a *p*-value of 0.05 or less was considered to indicate statistical significance.

## 3. Results

Thirty-six subjects (21 men, 15 women) with an average age of 48 ± 5 years, who were initially recruited for the study, completed both intervention phases, and were included in the final analysis.

### 3.1. RBC Membrane Lipid Composition at Baseline

**Figure 2:**
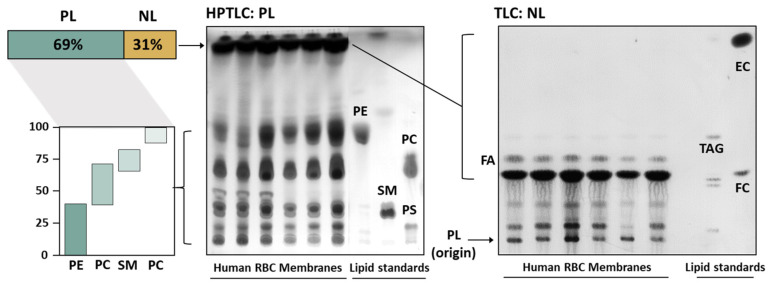
RBC membrane lipids from representative HPTLC and TLC plates. EC: Esterified cholesterol; FC: Free cholesterol; FA: Fatty acids; NL: Neutral lipids; PC: Phosphatidylcholine; PE: Phosphatidylethanolamine; PL: Phospholipids; PS: Phosphatidylserine; SM: Sphingomyelin and TAG: triacylglycerols.

As shown in Figure 2, the analysis of RBC membrane lipids by HPTLC showed that PL were the major lipid class representing the 69%, while NL (FC and FA) represented the 31% of the lipid membrane composition. Between PL fraction, PE was the major subclass, representing the 36% of the lipids, followed by PC, SM and PS (31, 21 and 12%, respectively). Furthermore, the study of NL by TLC, Figure 2, showed that FC was the major NL. In fact, along with PL, FC is one of the main structural components of erythrocyte membranes. Although TAG and esterified cholesterol (EC) are also found as components of some cell membranes, their solubility in phospholipid membranes was quite limited.

**Figure 3:**
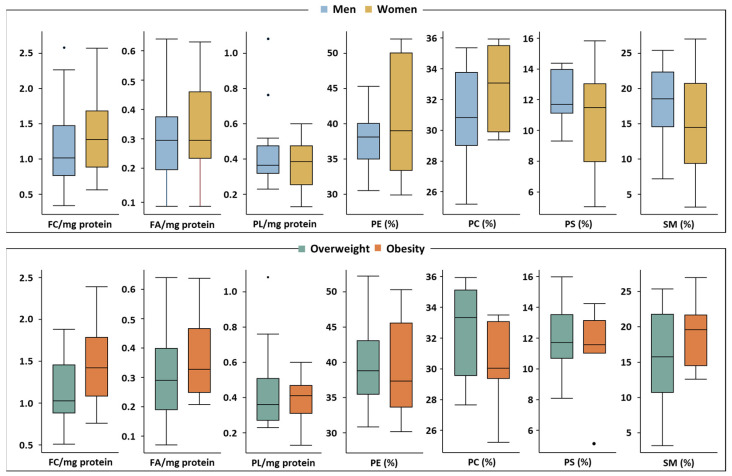
Sex-related and individual (overweight vs. obese) differences in lipid composition of erythrocyte membranes at baseline. Data are expressed as median (IQR). Differences between sexes and BMI were analyzed by Mann-Whitney test. No statistically significant differences were observed (*p* > 0.05). *n* = 36 for NL and *n* = 18 for PL subclasses.

Differences in the baseline lipid composition of erythrocyte membranes from the study volunteers were analyzed regarding sex and BMI (Figure 3) at baseline. Regarding sex-related differences a similar profile was observed between sexes for FC, FA and PL (*p* > 0.05). In addition, no statistically significant difference was observed for PE, PC, PS and SM between sexes (*p* > 0.05).

Differences in lipid composition were also assessed by BMI at baseline (Figure 3). No significant differences were observed on the membrane lipid profile (FC, FA, PL and PL subclasses) between volunteers with overweight and obesity at baseline (*p* > 0.05).

### 3.2. Effect of Beer Intake on RBC Membrane Lipid Composition

As shown in Table 1, alcohol free beer intake led a significant increase of FC (*p* < 0.008) and FA (*p* < 0.001). In addition, there was a tendency to increase the levels of PL (*p* = 0.062) and non-significant differences was observed for FC:PL ratio (*p* = 0.357). On another hand, traditional beer consumption during four-weeks, in addition to reaching higher amounts of FC (*p* < 0.007) and FA (*p* < 0.001), an increase was also observed for PL (*p* < 0.010). Regarding to FC:PL ratio there was a tendency to increase (*p* = 0.087). A comparative assessment of the differences between the two experimental groups indicated that while the changes observed in the cohort receiving the traditional beer were more pronounced, statistical analysis did not reveal any significant differences for FC, FA, PL, and FC:PL ratio (*p* > 0.05) (Table 1). In addition, not significant differences were detected for the fold changes between alcohol free beer group and traditional beer group (*p* > 0.05).

Regarding PL fraction, the consumption of non-alcoholic beer leads to an increase in the relative abundance of PE (2.73%) and a decrease in SM and PC (1.59 and 1.24%, respectively) (*p* < 0.05) after four-weeks of intake in overweight and obese volunteers (Table 1). On the other hand, four-weeks of traditional beer intake leads to a significant increase in the relative abundance of PE (3.05%) and a decrease in PC (1.74%) (*p* < 0.05). Regarding PS, in both groups this fraction remained unchanged (*p* > 0.05). In addition, several ratios were calculated (PC:PE, PC:SM, and PE:PS), however, significant differences were only observed in the PC:PE ratio in both intervention groups (*p* < 0.05). Comparison of the relative abundance for each of the PL subclasses showed that there was no significant difference in the changes observed between both interventions (*p* > 0.05) (Table 1). Additionally, the analysis of the ratios showed no statistically significant differences between groups (*p* > 0.05). Moreover, no statistically significant differences were observed in the fold changes between the free alcohol beer group and the traditional beer group for the PL subclasses (*p* > 0.05) (Supplementary Table S2).

When sex differences in the levels of RBC lipids at the end of the interventions were investigated, it was found that women showed a higher abundance of PC (*p* value = 0.031) after alcohol free beer intake (*p* value = 0.031), while no statistically significant differences were observed for the other lipids species (Supplementary Table S3). After 4 weeks of traditional beer consumption, lipid profiles were similar between women and men (*p* > 0.05). Additionally, the changes observed following the consumption of both alcoholic and non-alcoholic beer were similar between the sexes (*p* > 0.05).

The comparison of RBC lipids in function of BMI (overweight vs. obesity) was also evaluated (Supplementary Table S4). At the end of the intervention, obese individuals of the alcohol-free beer group presented a higher abundance of SM (*p* = 0.042) and FC/PL ratio (*p* = 0.010) and a lower relation of PC/SM (*p* = 0.034). In specific in this group, obese subjects showed a significant increase in SM and FC/PL ratio accompanied by a reduction PC/SM (*p* < 0.005), the opposite pattern was observed in overweight individuals. No significant differences were observed at the end of the intervention with traditional beer between overweight and obese individuals.

### 3.3. Metabolic Remodeling of the Human Red Blood Cell Membrane

**Figure 4:**
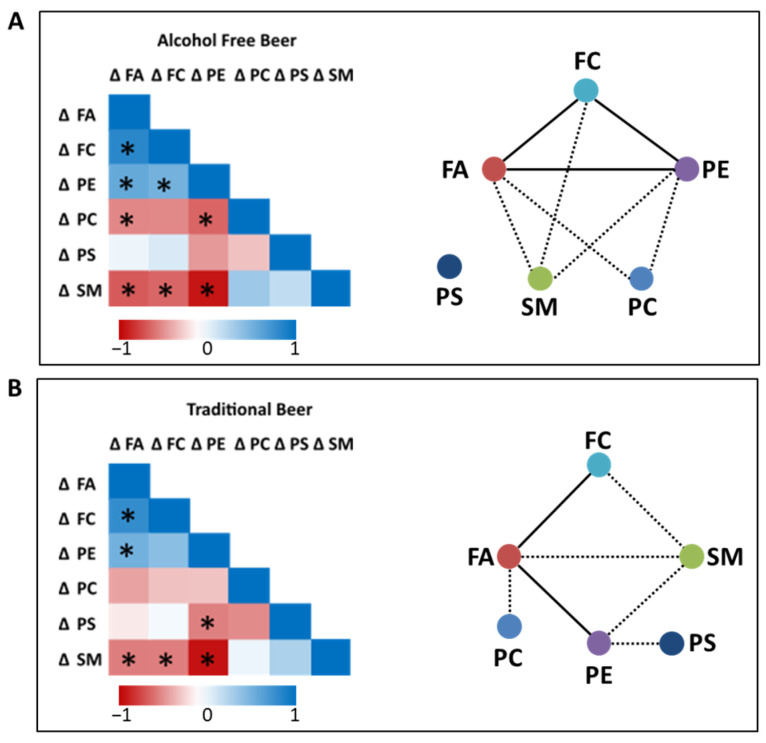
Heat maps and correlation networks of changes in NL and PL subclasses after 4-weeks of dietary intervention with alcohol-free beer (**A**) or traditional beer (**B**). In the pairwise correlation map, red shows a negative correlation and blue a positive correlation. Correlation networks were constructed based on their Spearman correlation coefficient. In each network, colored nodes refer to different lipid species and lines link correlated pairs. Dotted line means negative association. Line means positive association. * Means statistically significant difference, *p* < 0.05.

To better understand the relationship between the remodeling of the human RBCs membrane lipids we correlated (Pearson correlation) the changes observed for the NL and PL subclasses (Figure 4). In addition, correlation networks were constructed based on their Pearson correlation coefficient, demonstrating that the lipids were highly correlated with each other mainly in the free-alcohol beer group (Figure 4A,B).

In the non-alcoholic beer group, an increase in FA was positively associated with FC and PE (r > 0.6) and negatively correlated with PC and SM (r > −0.4). An increase in the FC levels were associated with higher levels of PE, while a decrease in FC levels were associated with an increase in the relative abundance of SM (r = 0.5462 and −0.5999, respectively). Regarding to PE, the higher the abundance, the lower the abundance of PC (r = −0.6018) and SM (r = −0.9147). PS represents the only lipid specie that was not related to any other lipid in the non-alcoholic beer group.

In regard with the associations in traditional beer group strong positive associations were detected between FA and FC and PE (r > 0.5) and strongly negative between FA and SM (r > 0.5). FC was associated negatively with SM (r = −0.5034). In addition, a very strong negative association between PE and SM was observed (r = −0.9287) and a moderate negative correlation with PS (r = −0.4957).

### 3.4. Associations between Membrane Lipids and Circulating Lipids

**Figure 5:**
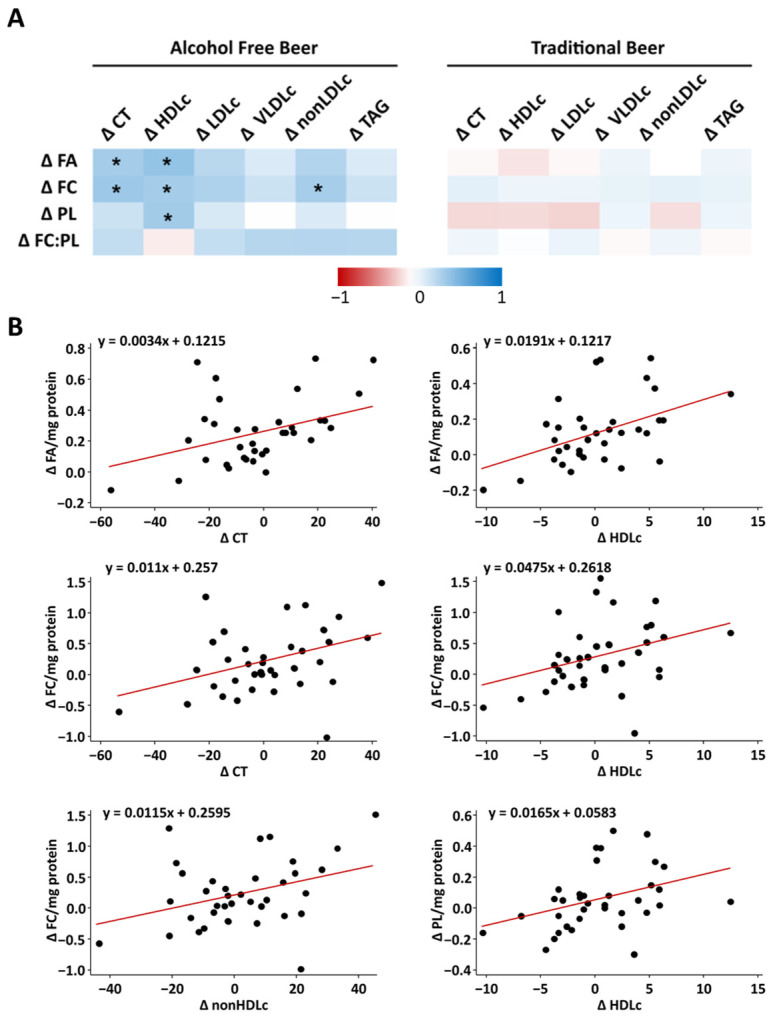
(**A**) Pearson correlation between the changes observed after 4-weeks of alcohol-free beer or traditional beer intake in NL of RBCs membranes and plasmatic lipids. In the pairwise correlation map, red shows a negative correlation and blue a positive correlation. (**B**) Bivariate scatter diagram only on those variables that showed a statistically significant correlation. * Indicates significance with Pearson correlation coefficient test (*p* < 0.05). Δ: Change.

Figure 5A show the Pearson correlations between the changes in FA, FC and PL of RBCs membranes and the changes observed for serum lipid profile. Compare with traditional beer group (where not significant associations were detected), several associations were detected in the alcohol-free beer group, all of them positives (*p* < 0.05). To be specific, an increase in FA was related with higher levels of total cholesterol (r = 0.355) and cholesterol transported for HDL (r = 0.424). At a higher concentration of FC, an increase in total cholesterol (r = 0.384), cholesterol transported by HDL (r = 0.354) and nonHDLc (r = 0.353) was also observed. Regarding PL, the more PL in the erythrocyte membrane the more HDL was observed (r = 0.369). In addition, a bivariate scatter plot is also shown only for those variables that show a statistically significant correlation (alcohol-free beer group, Figure 5B).

## 4. Discussion

Several observational studies and meta-analyses have consistently shown that moderate alcohol consumption is associated with protection against coronary artery disease and ischemic stroke [18,19]. We previously demonstrated that moderate beer intake (traditional and alcohol-free) does not exert vascular detrimental effects or increases body weight in obese healthy individuals [17]. In addition, we also demonstrated that regular moderate intake of traditional and alcohol-free beer attenuates the inflammasome signaling pathway in human macrophages [20]. Here we perform a prospective two-arm longitudinal crossed-over study to elucidate the effects of beer (alcoholic and non-alcoholic) intake on erythrocyte membrane lipid composition in overweight and obese volunteers. To our knowledge, this is the first study showing that beer intake (non-alcoholic or traditional) promotes changes in the lipid profile of erythrocyte membranes.

Regarding FA, in the study carried out for Pawlosky and Salem [21] demonstrated the effect of ethanol on essential FA metabolism. Stimulation of FA anabolism seems to be the mechanism by which low doses of alcohol intake may promote changes in FA profile (for example an increase in polyunsaturated fatty acids, PUFAs) [21]. On the contrary, when higher amounts of alcohol are consumed, the concentration of PUFAs decreases due to an increase in the FA catabolism [21]. This could explain why, although not significant, the changes in FA content were more evident in the group that had moderate consumption of traditional beer during four-week (0.20 vs. 0.13 FA/mg proteins). In a study carried out in 2009, alcohol intake (wine) was associated with higher concentrations of ω3-FA in RBCs membranes [22]. In the mentioned study, in addition to the alcohol effect on FA metabolism, it was also suggested that non-alcoholic components of wine, namely polyphenols, could also interact with the metabolism of essential PUFAs. Therefore, in our study, the synergistic effect of alcohol and polyphenols (which were in higher concentrations in traditional beer) could tentatively be related to the more pronounced changes in the group that had moderate consumption of traditional beer for 4-weeks. In addition, the changes observed in the RBCs membranes of the volunteers who received the alcohol-free beer may be related to the polyphenols in the beer that promoted the metabolism of FA.

The lipid composition of the cell membrane, particularly cholesterol, influences various functions of embedded enzymes, transporters, and receptors in RBCs. High membrane cholesterol content affects the RBCs’ main vital function, O_2_ and CO_2_ transport and delivery, with consequences on peripheral tissue physiology and pathology [14]. Additionally, it is well established that cholesterol in the cell membrane not only impairs transport processes but also affects the cell’s deformability [23]. Since erythrocyte membrane fluidity depends on (i) cholesterol content and the presence of other neutral lipids and (ii) phospholipid composition and phospholipid fatty acid pattern [24] according with our results, the daily intake of non-alcoholic and traditional beer during four weeks causes minimal changes in membrane fluidity in overweight and obese individuals. This finding is supported by the fact that, although daily intake of both non-alcoholic beer and traditional beer causes an increase in erythrocyte membrane FC, minimal changes in PL were observed, the increase being significant only in the group receiving traditional beer. In a study carried out in 2010, was demonstrated that chronic alcoholism causes alterations in the membranes, leading to an increase in total cholesterol and a tendency to decrease PLs [24]. Human evidence also suggests that erythrocyte membrane PL composition is affected by dietary fat and host factors (e.g., age, BMI, WHtR) [25]. Recently, was demonstrated that alcohol-induced lipid peroxidation causing an increase in PL in drinking male volunteers with regular practice of consuming alcohol every day for at least 70–80 g for the last 8–10 years [26]. In addition to changes in the PL fraction in erythrocyte membranes due to ethanol, the changes may also be due to the minority composition of beer. Certain biological activities of phenolic compounds are attributed to their interactions with the PL bilayer component found in membranes. These activities linked to the membrane involve specific interactions between lipids and phenolic compounds, such as preventing lipid peroxidation, as well as non-specific effects that modify the biophysical characteristics of the membrane [27].

In our study we observed that an increase in FC was associated with an increase in FA. Interesting, higher levels of FC were correlated with an increase in HDL in the free alcohol-beer group and with total blood cholesterol. Since RBCs, which carry large amounts of FC in their membrane, have been shown to play an important role in reverse cholesterol transport [28], it is not surprising that they are related to HDL levels. It is also not surprising that an increase in blood cholesterol levels is associated with higher levels of FC in erythrocyte membranes. It was demonstrated that approximately 50% of circulating cholesterol is carried in RBC membranes and that the magnitude of the cholesterol flux through RBCs is comparable to the total efflux of free cholesterol from tissues [29]. In our study a positive association between changes in PL and HDL was detected. In the EPIC-Norfolk Prospective Study [30] no overall significant relationship between total plasma PL concentration and coronary heart diseases was found. It is conceivable that the observed correlation can be ascribed to the intrinsic ability of HDL to serve as endogenous antioxidants, thereby exerting a protective effect against lipid oxidation occurring in biological membranes [31].

In addition, no alterations were observed in the FC/PL ratio in our study. In a study was suggested that an increase in cholesterol-to-PL ratio results in loss of membrane fluidity [32,33]. In specific, in the study carried out by Meurs and colleagues showed that a two-fold increase in FC/PL ratio decreased deformability and increased osmotic fragility, causing reduced lifespan of RBCs [34]. It has also been proposed that decreases in membrane fluidity are primarily in the membrane FC/PL range of 1.0 to 2.0; however, little additional change in fluidity occurs when the membrane FC/PL ratio increases to 2.0–3.0 [33]. According to the results obtained, the daily intake of beer with and without alcohol does not affect the initial RBC membrane fluidity of the study participants, given that the FC/PL ratio at the beginning of the intervention is greater than 3 in both groups and greater than 4 after the intervention.

Regarding PE, the most abundant PL subclass, an increase was observed after four weeks of beer intake (alcoholic and non-alcoholic) in overweight and obese volunteers. It is also important to note that the most profound changes were also observed in the group that received the traditional beer (without reaching significant differences between groups). To our knowledge, the effect of alcohol or polyphenol consumption on the PE of erythrocyte membranes has been scarcely studied. In a study with healthy male adults an increased in PE-monounsaturated fatty acids and PE-PUFAs in RBC was observed in average drinkers (30.2–63.4 g/day, essentially red wine) compared with non-drinking volunteers [35]. Although the mechanism (such as the activation of δ-6 and δ-5 desaturases) causing these alterations was not well defined. The fact that PE are good markers of dietary factors was also evidenced in healthy adults who showed changes in PE containing DHA in response to oral supplementation with DHA (510 mg DHA/day for 29 days) [36].

Additionally, the analysis of the PL fraction revealed a noteworthy reduction in the abundance of PC in both experimental groups following a four-week intervention period. The traditional beer group exhibited the most pronounced decrease in PC (−1.74 vs. 1.24%), without reaching statistical significance. Another important finding of our study was that women showed a higher abundance of PC than men at the end of the intervention with alcohol free beer. In research involving healthy adult males, it was noted that those who were moderate drinkers (consuming an average of 30.2–63.4 g/day, primarily red wine) showed an increase in PC rich in PUFAs compared to individuals who did not consume alcohol [35]. However, to our knowledge there are no studies that reveal the effect of moderate beer intake on erythrocyte membrane PCs. The divergent roles of choline metabolites in the pathogenesis of cardiometabolic risk factors and cerebrovascular diseases are well known. In a cross-sectional subset of the nutrition, aging and memory in elder’s cohort [37] higher plasma PC was associated with characteristics of both favorable (higher HDL and lower BMI). In our study the abundance of PC was not dependent on BMI (overweight vs. obesity) in both groups. The RBCs PC:PE ratio has been suggested as an indicator of presence of pro-inflammatory molecules in the blood, attributed to selective PC hydrolysis in the erythrocyte membrane [38]. In human studies, a decreased in PC:PE ratio has been linked to conditions such as obesity [39], non-alcoholic fatty liver disease [40], prediabetes, and type 2 diabetes [41]. In our study we have observed a minimal change in this ratio (−0.09, one-fold change) in both groups. Our results indicate that the moderate alcohol intake in traditional beer nor the alcohol-free beer induce a detrimental effect on RBCs lipid composition.

The relative abundance of SM was reduced in the alcohol-free beer group (1.61 ± 0.80%). High plasma levels of SM have been proposed as a marker of atherogenic remnant lipoprotein accumulation that may predict lipoprotein susceptibility to arterial wall sphingomyelinase [42]. In a human study it was suggested that increased levels of SM may play a role in plaque instability in acute coronary syndrome and may be the mechanism underlying elevated cholesterol levels in patients with coronary artery disease [43]. In our study, we did not detect a positive relationship between SM and FC levels. Beer is a beverage rich in phenolic compounds from hops (30%) and malt (70–80%) [44], being the phenolic acid and flavonoids, the main families identified [45]. The impact of flavonoids on the hydrophilic and hydrophobic regions of membranes was demonstrated in a biophysical study [46]. In our study, since the abundance of SMs was significantly reduced only in the group that received the non-alcoholic beer, this could be due to a protective effect of minority compounds such as polyphenols. Finally, no changes were observed in the relative abundance of PS.

It is crucial to emphasize the pronounced interplay existing among the diverse lipid species within the membranes of erythrocytes. This finding could suggest the role of diet in lipid metabolomics remodeling indicating that changes occur in a connected manner affecting all lipid species. PC and SM primarily reside in the membrane outer leaflet (facing the outside environment) while the inner leaflet (facing the inside of the cell) is known to mainly consist of PE and PS [1]. The detection of negative associations between the abundance of PC or SM and PE or PS is not surprising, considering that PL are asymmetrically distributed in the RBCs membrane. This observation implies a reciprocal relationship, wherein an increase in one lipid species coincides with a decrease in the other, likely attributable to their distinct functions and distribution across the membrane structure.

This study has several limitations in this exploratory clinical study. First, targeted lipidomic analysis was not performed; second, the sample size of the clinical study was small; and third, the results were not externally validated. After the changes observed in this exploratory study, future studies should use integrative untargeted and targeted metabolomics and lipidomic in RBCs to further evidence potential differences between the intervention groups.

## 5. Conclusions

In conclusion, our results demonstrate that beer consumption, both traditional and without alcohol, mildly modifies the lipid composition of RBC membranes. In specific, four weeks of alcohol-free beer consumption resulted in increased levels of FA, FC, and PE, along with a reduction in PC and SM. Meanwhile, traditional beer intake led to erythrocyte membranes with higher levels of FA, FC, PL, and PE, and lower PC levels. The changes observed were consistent across both groups. Another of our findings is that the observed alterations in membrane lipids were found to be independent of sex and BMI as influencing factors.

In summary, the lipid composition of erythrocyte membranes is distinctly but mildly influenced by the consumption of both non-alcoholic and conventional beer, with no effects on RBC membrane fluidity.

## Figures and Tables

**Table 1:** NL (mg protein) and PL (%) subclasses in RBCs membranes at the beginning of the intervention (Baseline) and after of four weeks of dietary intervention with alcohol free beer or traditional beer.

Lipids	Baseline	Alcohol Free Beer			Traditional Beer			*p*-Value ^2^
		After	Δ	*p*-Value ^1^	After	Δ	*p*-Value ^1^	
FA	0.32 ± 0.02	0.45 ± 0.03	0.13	<0.001	0.52 ± 0.05	0.20	<0.001	0.124
FC	1.27 ± 0.07	1.55 ± 0.09	0.28	0.008	1.58 ± 0.11	0.31	0.007	0.804
PL	0.40 ± 0.03	0.47 ± 0.04	0.07	0.062	0.50 ± 0.04	0.10	0.010	0.402
FC/PL	3.68 ± 0.38	4.27 ± 0.68	0.58	0.357	4.46 ± 0.72	0.68	0.087	0.932
PE (%)	39.53 ± 1.35	42.25 ± 1.32	2.73	0.026	42.58 ± 1.58	3.05	0.050	0.731
PC (%)	32.20 ± 0.67	30.95 ± 0.58	−1.24	0.019	30.46 ± 0.60	−1.74	0.003	0.383
PS (%)	11.69 ± 0.59	11.80 ± 0.58	0.10	0.784	11.65 ± 0.51	−0.04	0.686	0.750
SM (%)	16.58 ± 1.32	15.00 ± 1.44	−1.59	0.049	15.31 ± 1.46	−1.27	0.182	0.808
PC/PE	0.83 ± 0.03	0.74 ± 0.03	−0.09	0.014	0.74 ± 0.05	−0.09	0.023	0.731
PC/SM	2.33 ± 0.33	2.70 ± 0.51	0.37	0.126	2.64 ± 0.37	0.16	0.334	0.731
PE/PS	3.70 ± 0.41	3.85 ± 0.36	0.15	0.424	3.81 ± 0.28	0.12	0.951	0.951

Data are given as mean ± SEM. *n* = 36 for NL and *n* = 18 for PL subclasses. *p*-value ^1^: Comparison between baseline and final levels of each intervention were analyzed by paired *t*-test for NL and Wilcoxon-test for PL subclasses; *p*-value ^2^: Comparison of the changes observed at the end of the interventions between both groups were analyzed by paired *t*-test for NL and Wilcoxon-test for PL subclasses. *p* < 0.05 indicates significance.

## Data Availability

The information outlined in the manuscript, as well as the code book and analytic code, will be provided upon a reasonable request, subject to scientific approval.

## Supplementary Materials

The following supporting information can be downloaded at: https://www.mdpi.com/article/10.3390/nu16203541/s1, Table S1. Individual and total phenolic compound content of the beers administered to the volunteers. Table S2: Fold changes after four weeks of alcohol-free beer or traditional beer intake in overweight and obese individuals. Table S3. Comparison between sexes in the final levels and the changes observed after the consumption of beer, both alcoholic and non-alcoholic. Table S4. Comparison of final levels and changes observed between overweight and obese individuals following the consumption of both alcoholic and non-alcoholic beer.
