# Supplementary material for: Effect of Moderate Beer Intake on the Lipid Composition of Human Red Blood Cell Membranes
Source: Nutrients. 2024 Oct 18;16(20):3541. doi: 10.3390/nu16203541 (PMC11510343; doi:10.3390/nu16203541)
Supplement: Supplementary file 1 [file nutrients-16-03541-s001.zip › nutrients-3261902-supplementary.pdf]

Article

Effect of Moderate Beer Intake on the Lipid Composition of Human Red Blood Cell Membranes

Anallely López-Yerena [1], Natalia Muñoz-García [1], Victoria de Santisteban Villaplana [1,2], Teresa Padro [1,3,†] and Lina Badimon [1,3,4,†,*]

[1] Institut Recerca Sant Pau, Sant Antoni Mª Claret 167,08025 Barcelona, Spain; naye.yerena@gmail.com (A.L.-Y.); nmunoz@santpau.cat (N.M.-G.); vsantisteban@santpau.cat (V.d.S.V.); tpadro@santpau.cat (T.P.)
[2] School of Pharmacy and Food Sciences, University of Barcelona (UB), 08036 Barcelona, Spain
[3] Centro de Investigación Biomédica en Red Cardiovascular (CIBER-CV), Instituto de Salud Carlos III, 28029 Madrid, Spain
[4] Cardiovascular Research Chair, Universitat Autònoma de Barcelona (UAB), 08193 Barcelona, Spain
* Correspondence: lbadimon@santpau.cat
† These authors contributed equally to this work.

Table S1. Individual and total phenolic compound content of the beers administered to the volunteers.

Phenolic compounds	Alcohol Free Beer	Traditional Beer
Flavonoids		
Kaempferol-*O*-glucoside	2.5 ± 0.1	3.1 ± 0.2
Quercetin-3-*O*-glucoside	10.9 ± 0.4	18.7 ± 0.7
Epicatechin	6.5 ± 0.2	6.4 ± 0.2
Quercetin	4.3 ± 0.3	4.9 ± 0.2
Rutin	3.6 ± 0.3	5.0 ± 0.1
Catechin	49.5 ± 3.0	47.1 ± 1.0
Phenolic acids		
4-hydroxybenzoic acid	13.2 ± 1.0	23.7 ± 1.0
Caffeic acid	18.6 ± 1.0	21.6 ± 1.0
Chlorogenic acid	0.7 ± 0.0	3.3 ± 0.1
Ferulic acid	120.9 ± 5.0	166.5 ± 4.0
p-Coumaric acid	46.2 ± 2.0	70.5 ± 3.0
Protocatechuic acid	6.8 ± 0.3	11.2 ± 0.4
Sinapic acid	27.3 ± 1.0	38.7 ± 1.0
Vanillic acid	3.6 ± 0.3	8.5 ± 0.3
Isoxanthohumol	55.8 ± 8.0	165.6 ± 32.0
8-Prenylnaringenin	5.7 ± 1.2	9.8 ± 0.9
Total	375.9 ± 157.0	604.8 ± 190.0

Values are given as mean ± SD. Data are expressed as mg/L.

Table S2. Fold changes after four weeks of alcohol-free beer or traditional beer intake in overweight and obese individuals.

Lipids	**Alcohol Free Beer**	**Traditional Beer**	***p*-value**
FC	132 ± 0.10	1.32 ± 0.10	0.972
FA	1.66 ± 0.19	1.84 ± 0.21	0.455
PL	1.23 ± 0.09	1.28 ± 0.09	0.617
FC:PL ratio	1.20 ± 0.14	1.10 ± 0.06	0.520
PE	1.08 ± 1.31	1.08 ± 1.30	0.870
PC	0.96 ± 0.66	0.95 ± 0.66	0.374
SM	0.90 ± 1.40	0.93 ± 1.42	0.519
PS	1.02 ± 0.36	1.03 ± 0.48	0.859
PC:PE ratio	0.91 ± 0.03	0.89 ± 0.02	0.627
PC:SM ratio	1.13 ± 0.32	1.09 ± 0.30	0.588
PE:PS ratio	1.08 ± 0.40	1.11 ± 0.41	0.681

FA: Fatty acids; FC: Free cholesterol; PC: Phosphatidylcholine; PE: Phosphatidylethanolamine; PL: Phospholipids; PS: Phosphatidylserine and SM: Sphingomyelin. Fold changes comparison between groups were analyzed by unpaired Student's t -test. n = 36. $p < 0.05$ indicates significance.

Table S3. Comparison between sexes in the final levels and the changes observed after the consumption of beer, both alcoholic and non-alcoholic.

Lipids	Alcohol Free Beer			Traditional Beer			*p-value*	
	W	M	*p-value*	W	M	*p-value*	W	M
End of intervention								
FC	1.55 ± 0.12	1.55 ± 0.13	0.981	1.63 ± 0.17	1.54 ± 0.14	0.357	0.943	0.936
FA	0.44 ± 0.06	0.45 ± 0.04	0.757	0.55 ± 0.09	0.51 ± 0.07	0.931	0.395	0.751
PL	0.43 ± 0.05	0.49 ± 0.05	0.595	0.46 ± 0.06	0.53 ± 0.06	0.733	0.519	0.732
FC/PL	4.16 ± 0.51	4.35 ± 1.12	0.252	4.95 ± 1.38	3.89 ± 0.65	0.409	0.806	0.728
PE	42.88 ± 2.77	41.94 ± 1.51	0.750	43.08 ± 3.44	42.34 ± 1.75	0.820	0.937	0.843
PC	32.77 ± 0.96	30.05 ± 0.59	*0.031*	31.85 ± 0.60	29.77 ± 0.78	0.119	0.621	0.58
PS	12.09 ± 0.98	11.65 ± 0.75	0.964	12.45 ± 1.05	11.25 ± 0.56	0.422	0.937	0.469
SM	12.26 ± 2.94	16.36 ± 1.52	0.213	12.63 ± 3.05	16.65 ± 1.53	0.336	0.699	0.887
PC/PE	0.78 ± 0.05	0.73 ± 0.03	0.423	0.77 ± 0.07	0.72 ± 0.05	0.509	0.788	0.434
PC/SM	4.02 ± 1.38	2.05 ± 0.21	0.100	3.39 ± 0.84	2.03 ± 0.25	0.213	0.937	0.965
PE/PS	3.72 ± 0.49	3.92 ± 0.50	0.982	3.63 ± 0.52	3.90 ± 0.29	0.494	0.818	0.478
Changes								
FC	0.08 ± 0.06	0.16 ± 0.04	0.119	0.19 ± 0.09	0.22 ± 0.07	0.097	0.878	0.632
FA	0.09 ± 0.15	0.41 ± 0.12	0.162	0.17 ± 0.16	0.40 ± 0.14	0.563	0.309	0.808
PL	0.05 ± 0.04	0.07 ± 0.05	0.918	0.08 ± 0.06	0.11 ± 0.05	0.745	0.675	0.623
FC/PL	-0.34 ± 0.37	1.25 ± 1.03	0.202	0.45 ± 0.66	0.79 ± 0.42	0.751	0.806	0.784
PE	2.25 ± 2.26	2.96 ± 1.32	0.553	2.44 ± 2.09	3.36 ± 1.55	0.964	0.937	0.843
PC	-1.26 ± 0.84	-1.24 ± 0.61	0.892	-2.18 ± 0.39	-1.51 ± 0.63	0.385	0.513	0.514
PS	0.65 ± 0.43	-0.17 ± 0.51	0.221	1.01 ± 0.21	-0.57 ± 0.70	0.080	0.394	0.551
SM	-1.64 ± 1.62	-1.56 ± 0.84	0.964	-1.27 ± 1.81	-1.28 ± 1.13	0.964	0.589	0.755
PC/PE	-0.08 ± 0.06	-0.09 ± 0.04	0.666	-0.09 ± 0.05	-0.09 ± 0.04	0.910	0.916	0.81
PC/SM	0.93 ± 0.60	0.09 ± 0.15	0.280	0.31 ± 0.34	0.08 ± 0.19	0.632	0.589	0.744
PE/PS	-0.04 ± 0.39	0.25 ± 0.21	0.336	-0.13 ± 0.24	0.24 ± 0.48	0.291	0.818	0.478

Data are given as mean ± SEM. NL are expressed as mg of protein and PL subclasses in %. Comparison between groups were analyzed by Wilcoxon-test t. $p < 0.05$ indicates significance. PW: Women; M: men.

Table S4. Comparison of final levels and changes observed between overweight and obese individuals following the consumption of both alcoholic and non-alcoholic beer.

Lipids	Alcohol Free Beer			Traditional Beer			*p-value*	
	OW	O	*p-value*	OW	O	*p-value*	OW	O
End of intervention								
FC	1.47 ± 0.13	1.63 ± 0.13	0.467	1.46 ± 0.12	1.68 ± 0.17	0.330	0.903	0.910
FA	0.48 ± 0.05	0.41 ± 0.05	0.261	0.46 ± 0.06	0.58 ± 0.09	0.358	0.596	0.230
PL	0.45 ± 0.04	0.48 ± 0.06	0.981	0.44 ± 0.05	0.57 ± 0.07	0.205	0.788	0.324
FC/PL	3.62 ± 0.45	4.92 ± 1.28	*0.010*	4.93 ± 1.27	3.79 ± 0.63	0.707	0.832	0.485
PE	43.68 ± 1.24	39.41 ± 2.94	0.250	42.33 ± 1.84	42.98 ± 3.04	0.860	0.695	0.366
PC	31.34 ± 0.75	30.18 ± 0.91	0.348	30.42 ± 0.88	30.52 ± 0.74	0.948	0.459	1.000
PS	12.07 ± 0.68	11.24 ± 1.16	1.000	12.45 ± 0.63	10.38 ± 0.66	0.100	0.596	0.218
SM	12.91 ± 1.64	19.17 ± 1.98	*0.042*	14.79 ± 1.78	16.12 ± 2.66	0.791	0.525	0.628
PC/PE	0.72 ± 0.02	0.79 ± 0.07	0.538	0.74 ± 0.05	0.74 ± 0.07	0.877	0.439	0.561
PC/SM	3.22 ± 0.72	1.68 ± 0.21	*0.034*	2.40 ± 0.30	2.62 ± 0.80	0.596	0.608	0.470
PE/PS	3.77 ± 0.26	4.02 ± 1.01	0.422	3.50 ± 0.24	4.31 ± 0.49	0.211	0.639	0.445
Changes								
FC	0.24 ± 0.14	0.31 ± 0.14	0.809	0.25 ± 0.12	0.35 ± 0.18	0.644	0.702	0.403
FA	0.15 ± 0.05	0.10 ± 0.04	0.448	0.14 ± 0.06	0.26 ± 0.08	0.894	0.955	0.447
PL	0.08 ± 0.04	0.04 ± 0.05	0.557	0.07 ± 0.04	0.13 ± 0.06	0.701	0.864	0.395
FC/PL	-0.40 ± 0.30	1.56 ± 1.19	*0.010*	0.91 ± 0.58	0.41 ± 0.47	0.707	0.153	0.151
PE	4.09 ± 1.53	0.01 ± 0.54	0.103	3.79 ± 1.59	1.90 ± 1.93	0.479	0.928	0.836
PC	-1.54 ± 0.61	-0.65 ± 0.77	0.385	-2.19 ± 0.46	-1.01 ± 0.84	0.596	0.459	0.534
PS	0.09 ± 0.52	0.14 ± 0.49	0.947	0.11 ± 0.48	-0.29 ± 1.08	0.878	0.892	0.836
SM	-2.63 ± 0.96	0.50 ± 0.56	*0.025*	-1.70 ± 1.24	-0.60 ± 1.47	0.536	0.525	0.945
PC/PE	-0.12 ± 0.04	-0.01 ± 0.02	0.144	-0.12 ± 0.04	-0.05 ± 0.04	0.316	0.892	0.556
PC/SM	0.59 ± 0.33	-0.06 ± 0.09	*0.026*	0.18 ± 0.23	0.12 ± 0.25	0.912	0.229	0.945
PE/PS	0.31 ± 0.26	-0.16 ± 0.18	0.437	0.3 ± 0.22	-0.17 ± 0.78	0.659	1.000	0.836

Data are given as mean ± SEM. NL are expressed as mg of protein and PL subclasses in %. Comparison between groups were analyzed by Wilcoxon-test t. $p < 0.05$ indicates significance. OW: Overweight; O: Obese.
